# Editorial: Understanding human biology with iPSC derived cell types in the Era of CRISPR technology

**DOI:** 10.3389/fcell.2023.1351676

**Published:** 2023-12-22

**Authors:** Huinan Li, Laralynne Przybyla, Joel W. Blanchard

**Affiliations:** ^1^ Laboratory for Genomics Research, University of California, San Francisco, San Francisco, CA, United States; ^2^ Icahn School of Medicine at Mount Sinai, New York, NY, United States

**Keywords:** CRISPR, genome editing, IPSC, stem cell, CRISPR screen

The discovery and optimization of CRISPR (Clustered Regularly Interspaced Short Palindromic Repeats) technology has led to a new wave of functional interrogation of individual genes, as well as large-scale and even genome-wide genetic screening with various phenotypic readouts to identify gene candidates in disease-relevant biology applications. Human induced pluripotent stem cells have enabled generation of iPSC-derived cell types of interest that are relevant across many areas of developmental biology and disease biology investigation. However, the combination of these two technologies is not trivial. This Research Topic contains three reviews expanding the topics of CRISPR screens to understand neural development and diseases, summaries of considerations and applications when conducting screens using stem cell models, as well as the pros and cons of using organoids as a system for biological questions. The topic also includes original research using iPSC-derived alveolar epithelial cells to investigate the function of the *ACE2* gene.

One challenging but rewarding way of combining iPSC and CRISPR technology is to investigate neural development and disease biology. Ahmed et al. reviewed different CRISPR technologies that can be applied for genetic screens, including format and readout considerations. The review provides an overview of current examples of CRISPR screens using human iPSC-derived neural cultures to address questions around identifying drivers of neural cell fate specification, the fitness landscape of human neurons, the regulation of immune responses in glial cells, and identifying modifiers of host-virus interactions in the brain. Furthermore, the authors discussed combining techniques to investigate the genetic factors on neurodevelopmental and neurodegenerative disorders in detail, including autism, microcephaly, and C9orf72- associated neurodegeneration. Finally, the authors cautioned researchers when considering CRISPR screens to be mindful of the scale of the library, coverage, and choice of cells to be screened, and whether to conduct 2D or 3D screens in an arrayed or pooled format with appropriate readouts.

Another review by Shevade et al. discussed the rationale for using iPSC-derived cells over immortalized cell lines and primary cells, as well as over animal models. In addition, the authors discussed how CRISPR screening can be conducted using co-culture and organoid culture models, and outlined elements to consider for building robust iPSC models, including identifying relevant cell lines, optimizing differentiation protocols, and establishing adequate cell line controls. The authors then discussed different types of high-throughput screens and development of screening assays and readouts in iPSC-derived cells. Finally, the authors discussed applications of CRISPR/Cas screening in iPSC-derived cell types including cardiomyocytes, islets, neurons, microglia, and astrocytes.

CRISPR and iPSC technology can also be combined to investigate and target mechanisms of viral infection. Niwa et al. provide a timely illustration of this by demonstrating that SARS-CoV2 requires the ACE2 receptor to infect Alveolar epithelial cells. To achieve this, Niwa et al. knock out the expression of ACE2 in human iPSC. After confirming gene knockout, they differentiate the iPSC into Alveolar lung epithelial cells and culture them on a unique transwell platform that creates an apical and basolateral surface. They find that the SARS-CoV2 virus readily infects the wild-type epithelial cell but not the ACE2 knockout cells. Future studies could build off this system by performing CRISPR screens to identify additional genetic modifiers of SARS-CoV2 infection.

For iPSC-derived cell types of interest, there are even more sophisticated systems that better recapitulate *in vivo* environments. In Fernandes mini-review, organoids are discussed as complex systems, with characterization, methods for studying organoids as complex systems, and further considerations in terms of genome editing and manipulation of organoid systems.

Together, the reviews and studies described in these Research Topic articles cover many considerations and applications of functional genomics in stem cell models and complex biosystems, which will provide researchers with new ideas for utilizing and effectively combining iPSC and CRISPR technologies to uncover novel disease-relevant biological mechanisms and lead to discoveries of potential therapeutic targets and strategies.

